# tDCS and Robotics on Upper Limb Stroke Rehabilitation: Effect Modification by Stroke Duration and Type of Stroke

**DOI:** 10.1155/2016/5068127

**Published:** 2016-03-31

**Authors:** Sofia Straudi, Felipe Fregni, Carlotta Martinuzzi, Claudia Pavarelli, Stefano Salvioli, Nino Basaglia

**Affiliations:** ^1^Neuroscience and Rehabilitation Department, Ferrara University Hospital, 44100 Ferrara, Italy; ^2^Center of Neuromodulation, Spaulding Rehabilitation Hospital, Harvard Medical School, Boston, MA 02129, USA; ^3^School of Physiotherapy, University of Ferrara, 44100 Ferrara, Italy

## Abstract

*Objective*. The aim of this exploratory pilot study is to test the effects of bilateral tDCS combined with upper extremity robot-assisted therapy (RAT) on stroke survivors.* Methods*. We enrolled 23 subjects who were allocated to 2 groups: RAT + real tDCS and RAT + sham-tDCS. Each patient underwent 10 sessions (5 sessions/week) over two weeks. Outcome measures were collected before and after treatment: (i) Fugl-Meyer Assessment-Upper Extremity (FMA-UE), (ii) Box and Block Test (BBT), and (iii) Motor Activity Log (MAL).* Results*. Both groups reported a significant improvement in FMA-UE score after treatment (*p* < 0.01). No significant between-groups differences were found in motor function. However, when the analysis was adjusted for stroke type and duration, a significant interaction effect (*p* < 0.05) was detected, showing that stroke duration (acute versus chronic) and type (cortical versus subcortical) modify the effect of tDCS and robotics on motor function. Patients with chronic and subcortical stroke benefited more from the treatments than patients with acute and cortical stroke, who presented very small changes.* Conclusion*. The additional use of bilateral tDCS to RAT seems to have a significant beneficial effect depending on the duration and type of stroke. These results should be verified by additional confirmatory studies.

## 1. Introduction

Stroke is a common primary cause of motor impairments and disability. Only about 15% of those with initial complete upper limb paralysis after stroke recover a functional use of their affected arm in daily life [1, 2]. Greater intensity of upper extremity training after stroke improves functional recovery [3] as well as repetitive task training [4]. Motor practice, in turn, favors motor cortical reorganization, which is correlated with the degree of functional recovery [5]. Robotic devices for upper extremity rehabilitation after stroke have been shown to improve arm function [6–9]. They may enhance conventional motor therapy, increasing repetitions of well-defined motor tasks (massed practice) with an improvement of motivation due to the feedback of the device; they can be programmed to perform in different functional modalities according to the subject level of motor impairment. Robotic assistance may increase sensory inputs and reduce muscle tone with an overall improved patients' confidence in performing movements and tasks that, without assistance, might be frustrating or even impossible to achieve [10]. In the past decade, neuromodulation approaches have been proposed with the aim of optimizing stroke motor rehabilitation. Among these, transcranial direct current stimulation (tDCS) represents a noninvasive tool to modulate motor cortical excitability inducing a brain polarization through the application of weak direct electrical currents on the scalp via sponge electrodes [11]. Transient, bidirectional, polarity-dependent modifications in motor cortical excitability can be elicited: anodal stimulation increases it, whereas cathodal stimulation decreases it [12, 13]. Moreover, on a behavioral viewpoint, tDCS can promote skilled motor function in chronic stroke survivors [14].

After a stroke, changes in motor cortex excitability occur leading to an unbalanced interhemispheric inhibition [11], because the depression of the contralesional hemisphere on the affected one is not balanced by a similar level of inhibition of the lesional hemisphere onto the unaffected one. It has been hypothesized that this phenomenon represents a potential maladaptive process with detrimental effects on arm motor function [15]. On this basis, to increase paretic arm function, an “interhemispheric competition model” has been adopted in noninvasive brain stimulation stroke research [11, 16]. Specifically, researchers applied anodal tDCS over the affected primary motor cortex (M1) [14], cathodal stimulation over the unaffected M1 [17], or, more recently, a combination of the two stimulation paradigms through a bilateral tDCS montage [18]. How noninvasive brain stimulation effects are relevant when coupled with a peripheral stimulation as rehabilitative interventions is now well established [19]. So far, tDCS effects on motor learning and arm function in stroke population have been extensively addressed in recent systematic reviews and meta-analysis reporting mixed conclusions [20–24]. Indeed, the effectiveness and timing of these new rehabilitative techniques need to be defined by further investigations. We can hypothesize that tDCS primes motor cortex circuits, increasing motor cortex excitability that is sustained after a robot-assisted training [25]. Furthermore, the combination of these techniques enhances synaptic plasticity and motor relearning through long-term potentiation- (LTP-) and long-term depression- (LTD-) like phenomena on M1 [26].

The aims of this exploratory pilot study were twofold. Firstly, we wanted to test the effects of a bilateral tDCS montage combined with upper extremity robot-assisted training (RAT) compared to RAT alone on motor recovery, gross motor function, and arm functional use in a heterogeneous sample of stroke survivors. Secondly, we explored whether additional factors such as stroke duration and type could modify and also be predictors of tDCS and RAT response.

## 2. Methods

This double-blinded exploratory RCT pilot study (NCT01828398) has been reviewed by the Ferrara University Hospital Ethics Committees. Written informed consent was obtained before all procedures. Inclusion criteria were as follows: (i) age (18–75 y); (ii) diagnosis of first stroke (ischemic or hemorrhagic verified by brain imaging); (iii) upper limb motor impairments verified by Fugl-Meyer Assessment-Upper Extremity (FMA-UE); (iv) trunk control defined as a score >50 on the Trunk Control Test (TCT) [27]; (v) adequate understanding of verbal and written information, sufficient to complete the tests. Exclusion criteria were as follows: (i) impaired cognitive functioning (score less than 24 on the Mini Mental Status Examination); (ii) intracranial metal implants that can be stimulated, incorrectly positioned, or overheated by the electric current; (iii) other neurological or psychiatric disorders; (iv) severe cardiopulmonary, renal, and hepatic diseases; (v) pregnancy. Patients enrolled were randomized in blocks of 4, stratified by the time distance from stroke (subacute: <6 months; chronic phase: >6 months), using a program available online (http://www.randomization.com/). They were allocated into two different treatment groups: upper extremity robot-assisted training + real-tDCS (experimental group) or upper extremity robot-assisted training + sham-tDCS (control group). Every patient received five sessions/week (Mon-Fri) over two weeks (10 sessions).

### 2.1. Transcranial Direct Current Stimulation

The anode was placed on the M1 of the affected hemisphere and the cathode on the contralateral M1 area. Electrodes were located at C3 and C4 according to the 10/20 international EEG system. The goal of this montage is to decrease cortical excitability in the unaffected motor cortex and increase it in the affected motor cortex as demonstrated before [28]. The direct current was delivered through a pair of sponge electrodes with a surface of 35 cm^2^ (7 × 5), soaked in saline solution. It was generated by a constant current stimulator, with rechargeable batteries (Brainstim, EMS, Italy). This continuous stimulation lasted 30 minutes, with an intensity of 1 mA during RAT. For sham condition tDCS, current was delivered for only 30 seconds and then the current was discontinued, but the tDCS apparatus was left in place for the same time as active tDCS (30 minutes). This procedure has been suggested as an effective blinding method in parallel clinical trials of tDCS [29, 30].

### 2.2. Upper Extremity Robot-Assisted Training

A robotic end-effector device has been used for the training protocol (REO Therapy System, Motorika, LTD, Israel). It consisted of a telescopic arm connected to a portable monitor, with software that allows multiplanar reaching training of the proximal upper limb in passive mode, guided, free, and against resistance. Specifically, shoulder flexion-extension and abduction-adduction combined with elbow flexion-extension were trained. Each session lasted about 30 minutes. The efficacy of this device has been explored in a noncontrolled trial in chronic stroke survivors [8].

### 2.3. Outcome Measures

Outcome measures were assessed the week before treatment initiation (T0) and the week after the end of treatment (T1) by a researcher blinded to the treatment received. It is important to point out that the investigator administering tDCS was not the same as the investigator assessing the outcomes. The Fugl-Meyer Assessment-Upper Extremity (FMA-UE) scale was performed (score ranges from 0 to 66) to assess arm motor recovery [31]. This measure has been considered suitable to detect changes in motor recovery in stroke survivors [32]. The Box and Block Test (BBT) was used to evaluate gross motor function. It counts the number of blocks that can be transported from one compartment of a box to another within 1 minute [33]. A semistructured interview, the Motor Activity Log (MAL), was administered to quantify real-world arm use in activities of daily living. Patients were asked to rate Quality of Movement (QOM) and Amount of Movement (AOM) during 14 tasks that include object manipulation as well as the use of the arm during gross motor activities. Each item is scored on a 6-point ordinal scale [34]. A tDCS side effects questionnaire (headache, neck pain, burning, redness, and/or itching at the site of stimulation) was administered after each session.

### 2.4. Statistical Analysis

Primary outcome measure was motor recovery measured by FMA-UE. Descriptive statistics (mean, standard deviation, and median) were used at T0 and T1. Baseline characteristics and clinical tests were compared between groups using the Mann-Whitney test or Pearson's chi-square test. Wilcoxon matched-pair signed-ranks test was performed to investigate time effects (T0 and T1) within groups; *z*-score has been reported for significant results. Mann-Whitney test was used to test differences among groups. We then examined the effects of covariates on our results by conducting subgroup analysis using a linear regression model. It was used to determine the effects of patients' sex and age and stroke characteristics (recovery stage, stroke location, side of the affected hemisphere, and ischemic/hemorrhagic stroke) on motor recovery improvement (T1-T0 FMA-UE score). A three-way ANOVA model (factors: treatment, recovery stage, and stroke location) was run to detect any possible interactions between predictors and treatment effects as to test for the potential effect modification of these variables. Statistical analysis was performed using STATA 13.1 software. Statistical significance was set to *p* < 0.05.

## 3. Results

**Figure 1:**
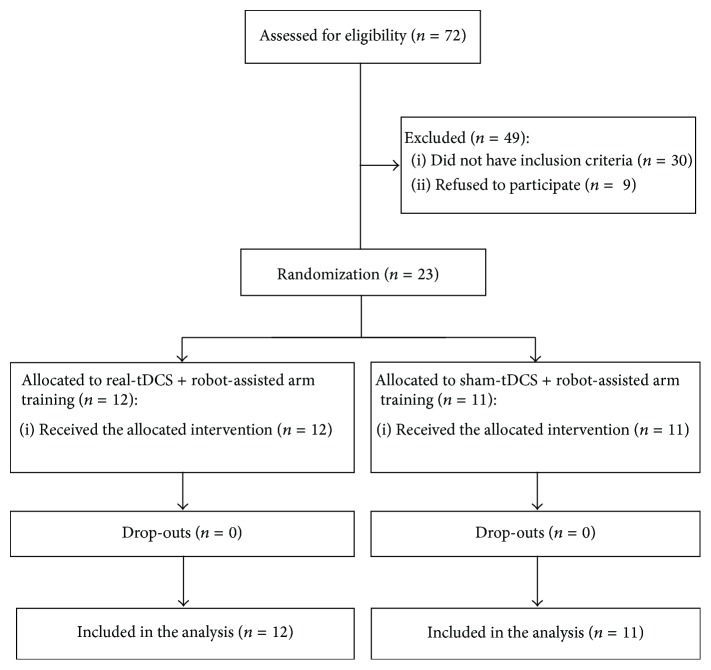
CONSORT study flow diagram.

We enrolled 23 stroke survivors; 12 were allocated to real-tDCS + RAT group and 11 to sham-tDCS + RAT group. The flow diagram of the study is reported in Figure 1.

Demographic, stroke, and functional baseline characteristics are summarized in Table 1. The two groups were similar in demographics (sex) and functional (FMA-UE, BBT, and MAL) and stroke parameters (onset, rehabilitation phase, stroke etiology, lesion type, and side hemisphere), except for age (*p* = 0.03).

In our initial analysis of comparing differences between active and sham-tDCS groups (univariate analysis), we found no significant differences. We then performed adjusted analysis and also tested for the interaction effects so as to test for potential effect modifiers. The effects of demographic and stroke characteristics on motor recovery were explored. Sex, stroke etiology, and side of the affected hemisphere were not predictors of motor improvements in our sample. Conversely, recovery stage in sham-tDCS group (*F* = 9.20, df = 1,9; *p* < 0.05; adjusted *R* ^2^ = 0.45) and stroke location in real-tDCS group (*F* = 8.48, df = 1,10; *p* < 0.05; adjusted *R* ^2^ = 0.40) were confirmed as predictors of motor recovery by a linear regression approach. A three-way ANOVA confirmed a significant main effect of recovery stage on motor function (*p* < 0.01) and a significant interaction effect (*p* < 0.01) of treatment (real- and sham-tDCS) and stroke location (subcortical and cortical). Treatment and recovery stage interaction effect was close to reaching statistical significance (*p* = 0.10). Based on these findings, a 3-point composite variable was created considering recovery stage and stroke location: patients were grouped as chronic subcortical stroke (*n* = 6), subacute subcortical or chronic cortical stroke (*n* = 11), or cortical subacute stroke (*n* = 6). A positive interaction between received treatment and this composite variable has been shown to be significant (*p* < 0.05). Post hoc analysis, considering motor recovery, revealed that there were significant differences in FMA-UE in both groups (real-tDCS group: *z* = −2.95, *p* = 0.003; sham-tDCS group: *z* = −2.80, *p* = 0.004). Gross motor function (BBT) and real-world arm functional use (MAL) were improved only in real-tDCS group (BBT: *z* = −2.29; *p* = 0.02; MAL-AOM: *z* = −2.21, *p* = 0.02; MAL-QOM: *z* = −2.21, *p* = 0.02). No between-group differences were highlighted among all outcome measures (see Table 2).

### 3.1. tDCS Adverse Effects Questionnaire

10 out of 23 patients reported mild side effects after stimulation (7 in the real-tDCS group and 3 in the sham-tDCS group): skin redness under the site of stimulation (6 : 5 in the real-tDCS group, 1 in sham-tDCS group), headache (2 : 1 in real-tDCS group and 1 in sham-tDCS group), sleepiness (1 in real-tDCS group), and neck pain (1 in sham-tDCS group).

## 4. Discussion

This is an exploratory pilot study where we applied bilateral tDCS combined with upper extremity robot-assisted training in a sample of stroke survivors. We highlighted an overall improvement in motor function, measured by FMA-UE, gross manual dexterity, measured by BBT, and functional use of the paretic arm in daily life, measured by MAL-AOM and QOM, lacking demonstration of any superiorities of real-tDCS on the sham-tDCS group. Possible explanation is that our sample was too small and heterogeneous (FMA-UE baseline: range 5–58). The lesion location and the tract-specific injury [35] have been related to arm impairment severity [36] and robotic treatment gains [35] more than the infarct size [36]. Therefore, 2/3 of our sample was based on cortical stroke (14/23) with a wide range of lesion size, a moderate to severe motor function, and a poor use of the affected arm (22 points FMA-UE and 0.63 MAL score at baseline) as the usual candidates to robot-assisted therapy [37]. Inconclusive findings may be explained even by patients peculiarities: in someone, a ceiling effect on motor recovery is reached by motor training alone, thereby making it impossible to detect further gains due to stimulation; in others with the brain in a state of maximal capability, low chance to increase cortical activity and motor performance is present [38]. Two other studies tested the combination of robot-assisted arm training and tDCS based on the “interhemispheric competition model” in severely impaired subacute [37] and chronic stroke survivors [39]. Hesse et al. did not evidence any superior effects of either anodal or cathodal tDCS compared to sham condition [37], whereas Ochi et al. found a significant but limited improvement in FMA-UE after both anodal tDCS and cathodal tDCS [39]. Conversely, positive results on arm motor recovery in low functioning stroke survivors have been reported by Wu et al. that applied cathodal tDCS on the affected M1 with the aim of reducing arm muscle tone [40]. So far, how the “interhemispheric competition model” failed to induce functional gains in severely impaired stroke survivors and that “no one size fits all” in stroke neuromodulation approach is recognized [41, 42]. O'Shea et al. found out how patients with a better motor function, measured by FMA-UE, showed greater improvement after cathodal tDCS compared with patients with poor arm recovery [41]. Yao et al. pointed out that reaching performance was reduced in low functioning patients after cathodal tDCS compared to mild stroke [43]. Bradnam et al. tested cathodal tDCS on proximal motor control and they showed how its effects strictly depend on the severity of the lesion. They postulated how, in severe stroke, the contralesional hemisphere plays a role in paretic proximal motor control [42]. In conclusion, these negative effects of cathodal tDCS on the unaffected hemisphere can be explained hypothesizing that more severe patients need a bilateral cortical activation to recruit volitional arm movements, as postulated by the “vicarious model” [44].

Early after stroke, a bilateral activation pattern in both the ipsilesional and contralesional hemispheres occurs [44]; whether such bilateral activation is adaptive or maladaptive is still in debate, even though a rebalance between hemispheres is considered a sign of good recovery in chronic phase [45]. In a chronic phase, persistent contralesional M1 activation seems to be related to impaired motor function through the mechanism of increasing interhemispheric inhibitory drives toward ipsilesional M1 during motor tasks [15]. Even though a clear correlation between neurophysiological and neuroimaging findings and motor outcome in stroke survivors is not entirely established, algorithms to predict motor recovery in subacute phase have been postulated [46]. Furthermore, in chronic stroke survivors, correlations between structural motor cortex connectivity and motor impairment [47] or cortical activation in ipsilesional primary and premotor cortex and good upper limb recovery [48] have been highlighted. On this basis, the “bimodal balance recovery model” tries to combine the “interhemispheric competition model” and the “vicarious model” taking into account a new variable, the structural reserve, which should determine which neuromodulation approach is more suitable [49]. Specifically, if the individual structural reserve is high, an interhemispheric competition approach is useful, whereas if it is low a vicarious approach will lead to more functional gains.

In our exploratory analysis, patients' response to bilateral tDCS was best predicted by a composite variable that reflected recovery stage (subacute or chronic) and site lesion (subcortical or cortical). The regression model showed that bilateral tDCS was more suitable for chronic stroke subjects with a subcortical lesion and was less effective for patients in a subacute phase after stroke or with a cortical stroke. Specifically, we found a bilateral tDCS larger effect size in the chronic subcortical subgroup (*n* = 6, ES = 1.47; CI = −0.55–3.37). Also, it needs to be underscored that other variables may also play an important role in the effects of tDCS on motor recovery in stroke and further studies should attempt to explore the effects of other variables. These findings are in line with previous studies that reported positive effects of bilateral tDCS on chronic stroke survivors when combined with constrained induced movement therapy [50] or with a meta-analysis that highlighted better tDCS results in chronic stroke population [20–22]. Conversely, in an acute stage, no bilateral [51], anodal [52], or cathodal [53] montage was found to be effective in restoring motor function, even if any was considered to be safe. Several reasons may explain differences among recovery stages: firstly, in the acute-subacute phase, the enhanced excitability of the intact hemisphere can be compensatory [44] rather than maladaptive; secondly, in this stage, neuromodulation effects may be masked by spontaneous recovery with too many confounders; thirdly, early after stroke, motor training can induce cortical reorganization several weeks after the end of treatment, suggesting that early motor training after stroke can help the evolving poststroke neural network [54]. Regarding brain stroke localization, our findings are in line with the previous meta-analysis that found a larger effect size in subcortical stroke [55].

No major adverse effects have been reported after tDCS sessions; only mild side effects as skin redness, headache, sleepiness, or neck pain were equally distributed among real and sham conditions. This is in line with a previous study on healthy and stroke patients [56] and also with a meta-analysis of stroke studies [55].

This exploratory study has several limitations that future studies should adequately address. Firstly, the small and heterogeneous sample size reduced the possibility to detect tDCS main effects. Future clinical trials matched for demographic and stroke characteristics are justified. Secondly, the absence of motor cortical excitability or brain resting state measures did not allow studying the effects on cortical reorganization processes. Thirdly, a long-term follow-up would be necessary to assess the motor skill learning retention phase that can be positively influenced by tDCS [21]. Fourthly, we combined bilateral tDCS during robot-assisted arm training: even if no difference has been found in delivering stimulation before, during, or after intervention [21], recent experiments suggested that doing stimulation before rehabilitation gives better results [57, 58]. Fifthly, even if age is not clearly correlated with motor gains after RAT [59] or tDCS [55] in stroke survivors, our results have to be taken cautiously considering that the two groups differ significantly by age (*p* = 0.03) as a consequence of a small sample size study. Lastly, in this pilot study, we combined tDCS with an end-effector device that trains only the proximal portions of the upper limb [8]; in future studies, task-oriented robot-assisted device that trains even the hand and fingers should be tested [60].

Furthermore, other neurophysiological techniques that induce neuroplastic changes after stroke have to be tested, such as paired associative stimulation (PAS) [61, 62] or brain-computer interface (BCI) [63].

## 5. Conclusions

In a convenience sample of stroke survivors, the bilateral tDCS approach combined with upper extremity robot-assisted therapy seems to be more effective in a chronic stage of recovery and patients with subcortical lesions.

## Figures and Tables

**Table 1:** Clinical and demographic characteristics.

	Real-tDCS + RAT (*n* = 12)	Sham-tDCS + RAT (*n* = 11)	Total (*n* = 23)	*p* value
Age (years)				
Mean (SD)	52.7 (16.0)	64.3 (9.7)	58.2 (14.4)	0.03
Median	58	67	66	
Sex (M/F)	5/7	7/4	12/11	0.29
Stroke etiology (ischemic/hemorrhagic)	10/2	9/2	19/4	0.28
Affected hemisphere (right/left)	3/9	5/6	8/15	0.30
Stroke type (subcortical/ cortical)	3/9	6/5	9/14	0.15
Stroke onset (weeks)				
Mean (SD)	40.7 (35.1)	78.2 (61.9)	58.6 (52.2)	0.22
Median	26	108	48	
Subacute stroke (<6 months)	5	4	9	—
Chronic stroke (>6 months)	7	7	14	—
FMA-UE baseline				
Mean (SD)	24.08 (16.60)	21.45 (13.23)	22.83 (14.85)	0.73
Median	20	17	18	
BBT baseline				
Mean (SD)	10.42 (15.47)	6.55 (11.67)	8.56 (13.62)	0.60
Median	2.5	0	0	
MAL-AOM baseline				
Mean (SD)	0.67 (0.9)	0.59 (1.02)	0.63 (0.94)	0.70
Median	0.28	0.15	0.21	
MAL-QOM baseline				
Mean (SD)	0.69 (1.01)	0.59 (1.17)	0.64 (1.07)	0.77
Median	0.14	0.15	0.14	

tDCS = transcranial direct current stimulation; *n* = number; SD= standard deviation; M/F = male/female; FMA-UE = Fugl-Meyer Assessment-Upper Extremity; BBT = Box and Block Test; MAL = Motor Activity Log; AOM = Amount of Movement; QOM = Quality of Movement; *p* value = difference between real-tDCS + robot-assisted arm training group and sham-tDCS + robot-assisted arm training group.

**Table 2:** Functional tests results (FMA-UE, BBT, and MAL) with significances.

	Real-tDCS + RAT (*n* = 12)		Sham-tDCS + RAT (*n* = 11)		*p* value
	Mean (SD)	Median	Mean (SD)	Median	
FMA-UE					
Pre	24.08 (16.60)	20	21.09 (13.19)	17	
Post	28.5^*∗∗*^ (18.96)	23	26.64^*∗∗*^ (16.12)	22	
Δ pre-post	5.17 (4.30)	4.5	5.5 (4.97)	5	0.82
BBT					
Pre	10.42 (15.47)	2.5	6.55 (11.67)	0	
Post	12.67^*∗*^ (17.23)	3.5	8.55 (14.07)	1	
Δ pre-post	2.25 (3.05)	1.5	2 (8.39)	0	0.057
MAL-AOM					
Pre	0.68 (0.90)	0.28	0.59 (1.02)	0.15	
Post	1.09^*∗*^ (1.36)	0.32	0.89 (1.38)	0.43	
Δ pre-post	0.41 (0.73)	0	0.3 (0.67)	0	0.61
MAL-QOM					
Pre	0.69 (1.01)	0.14	0.59 (1.17)	0.15	
Post	1.05^*∗*^ (1.43)	0.25	0.85 (1.50)	0.29	
Δ pre-post	0.36 (0.72)	0	0.26 (0.61)	0	0.51

tDCS = transcranial direct current stimulation; RAT = robot-assisted training; SD = standard deviation; FMA-UE = Fugl-Meyer Assessment-Upper Extremity; BBT = Box and Block Test; MAL = Motor Activity Log; AOM = Amount of Movement; QOM = Quality of Movement; Δ pre-post = changes between posttreatment and baseline; *p* value = difference between mean changes from baseline of real-tDCS + robot-assisted arm training group versus sham-tDCS + robot-assisted arm training group. *∗* = *p* < 0.05 or *∗∗* = *p* < 0.01 within-group differences in pre-post treatment.
